# Obsessive-compulsive symptoms, perceived burdensomeness, and thwarted belongingness: Associations and implications among US veterans

**DOI:** 10.1002/jclp.23609

**Published:** 2023-10-14

**Authors:** Tapan A. Patel, Amanda M. Raines, Danielle M. Morabito, Norman B. Schmidt

**Affiliations:** 1 Department of Psychology, Florida State University, Tallahassee, Florida, USA; 2 Southeast Louisiana Veterans Health Care System, New Orleans, Los Angeles, USA; 3 Louisiana State University School of Medicine, New Orleans, Los Angeles, USA

**Keywords:** obsessive-compulsive disorder, perceived burdensomeness, suicide, thwarted belongingness, veterans

## Abstract

**Objective:**

Obsessive-compulsive disorder (OCD) is among the most debilitating psychiatric disorders worldwide, but has gone relatively unnoticed within the US veteran population. Simultaneously, suicide rates continue to remain high within this population despite the high volume of veterans who receive psychiatric care. With recent research demonstrating OCD’s unique relationship with suicidality, it is imperative to explore this association and factors that may explain this association within veterans.

**Methods:**

The present study investigated OCD symptoms and their relationship with two known risk factors of suicide, perceived burdensomeness (PB) and thwarted belongingness (TB), in two samples of veterans.

**Results:**

In the first study (*N* = 100), OCD symptoms were found to be uniquely related to both PB and TB even after covarying for demographics, trauma exposure, and probable depression. In the second study (*N* = 99), these relationships were replicated longitudinally. OCD symptoms at baseline were found to be indirectly related to suicidal ideation severity at a 1-month follow-up via PB and TB at post-treatment.

**Conclusion:**

This study highlights the importance of assessing and addressing OCD symptoms within veterans due to the unique relationship these symptoms have with suicidal constructs. A deeper understanding of the impact of OCD within the veteran population will inform future prevention and intervention efforts.

## 1 | INTRODUCTION

Estimated to effect around 2.3% of the general US population (lifetime prevalence) (Ruscio et al., 2010), obsessive-compulsive disorder (OCD) is among the most debilitating psychiatric conditions worldwide. OCD is characterized by recurrent intrusive thoughts, images, or urges coupled with repetitive behaviors or mental acts aimed at reducing or alleviating the associated anxiety (American Psychiatric Association, 2022). OCD is associated with reduced quality of life and significant functional and occupational impairment (Huppert et al., 2009; Macy et al., 2013; Markarian et al., 2010; Murray & Lopez, 1996), highlighting the need for continued research in this area. Despite increased empirical attention in recent years, less is known however about OCD in certain subpopulations who may have increased risk of developing the disorder including military personnel and veterans.

Notably, the lifetime prevalence of OCD among veterans has been found to vary widely with ranges from 0.04% to 28% in studies using diagnostic interviews within the Veterans Health Administration (VHA) (Gros et al., 2013; McIngvale et al., 2019; Orsillo et al., 1996). These elevated rates of OCD among veterans have largely been attributed to the increased risk of trauma exposure that veterans experience compared to civilians (Kimbrel et al., 2015). Despite the relatively higher prevalence among veterans and relationship with trauma, OCD often goes unrecognized and untreated within this population (Glazier et al., 2013, 2015; Stanley et al., 2017). Indeed, many of the veterans who are diagnosed with OCD never receive treatment, and those that do have an average time to treatment ranging between 9 and 11 years (Marques et al., 2010). This lack of recognition could be attributable to the focus on treating depression and trauma-related disorders within this population (Kimbrel et al., 2015). Nevertheless, due to the functional consequences of OCD, it is vital to better understand the impact of these symptoms within veteran populations.

Failing to recognize OCD among veterans is particularly concerning considering that OCD is strongly related to suicide (Raines et al., 2014); for example, one systematic review found that severity of OCD was predictive of greater suicide risk (i.e., both ideation and attempts) along with severe anxiety, depression, hopelessness, and prior history of suicide attempts (Albert et al., 2019). Although OCD has been found to significantly increase the odds of suicidal ideation among civilians (odds ratio [OR] = 1.9–10.3), the relationship between OCD and suicidal ideation has remained largely unexplored among veterans (Albert et al., 2019; Schriver et al., 2020). Additionally, this lack of research is concerning given veterans are at increased risk of experiencing suicidal ideation, attempting suicide, and dying by suicide with recent reports stating that there are approximately 17 veteran suicide deaths per day (Department of Veterans Affairs, 2021).

In addition to a lack of studies investigating the relationship between OCD and suicidal thoughts and behaviors among veterans, there is a dearth of research evaluating the relationship between OCD and known psychiatric risk factors for suicide (e.g., anhedonia, impulsiveness, nonsuicidal self-injury; Nock et al., 2012; Patel et al., 2021). Two important risk factors to explore in relation to OCD and suicidal ideation are thwarted belongingness (TB; i.e., state when need for connectedness is not met) and perceived burdensomeness (PB; i.e., feeling one is a burden to others). According to the interpersonal theory of suicide, suicidal desire emerges when one believes they are a burden on others (i.e., PB), believes they are not connected to others (i.e., TB), and experiences hopelessness about these states (Van Orden, et al., 2010). Consistent with this theory, PB and TB have been associated with suicidal ideation and behavior in a variety of populations including rural and veteran samples (Compton et al., 2021; Nsamenang et al., 2013). However, less is known about the relationship between OCD, PB, and TB. Considering PB and TB are potentially modifiable risk factors for suicidal behaviors (Short et al., 2020), it is essential to understand how symptoms of OCD may be related to both. Importantly, prior research has found that OCD is related to loneliness and decreased social support, which could be considered analogs of PB and TB, respectively (Edwards et al., 2023; Timpano et al., 2014).

To this end, the purpose of the present investigation was to examine the relationship between OCD symptoms and PB and TB respectively within two veteran samples. In the first study, we evaluated the cross-sectional relationship between OCD symptoms and PB and TB after covarying for relevant demographic (i.e., sex assigned at birth and race, age) and clinical factors (i.e., trauma exposure and probable depression). In the second study, we examined the longitudinal relationship between OCD symptoms and suicidal ideation as well as the indirect effects of PB and TB in this relationship using a sample of veterans who participated in a treatment study for risk factors for suicide. In Study 1, we hypothesized that OCD symptoms would be uniquely and positively associated with both PB and TB after covarying for demographic variables as well as trauma exposure and probable depression. In Study 2, we hypothesized that OCD symptoms would be longitudinally associated with increased PB, TB, and suicidal ideation. Further we hypothesized that the OCD symptoms at baseline would be indirectly related to increased suicidal ideation at 1-month follow-up via PB and TB at post-treatment while covarying for trauma exposure and major depressive disorder.

## 2 | STUDY 1 METHODS

### 2.1 | Participants and procedures

The sample included 100 veterans receiving mental health services at a VHA rural community-based outpatient clinic (CBOC)^1^ in the southeast United States between 2017 and 2019. To assist with diagnostic clarification, treatment planning, and monitoring, veterans completed a brief battery of self-report questionnaires. Given that data were collected as part of routine clinical care, informed consent for research was not obtained. However, the VHA Institutional Review Board approved the use of these data for research purposes.

The sample was primarily male (80%) with ages ranging from 21 to 75. Most of the sample identified as Black/African American (52%), followed by White/Caucasian (42%), and other (e.g., biracial; 5%), with one veteran not responding (1%). Regarding marital status, 58% identified as married, 27% as divorced/separated, 13% as single, and 2% widowed. The majority of the sample served in the Army (59%), followed by Navy (17%), Marines (9%), National Guard (8%), and Air Force (5%), with 2% serving in more than one branch. Further, most served in combat operations in Vietnam (37%), followed by Iraq/Afghanistan (28%), other (e.g., peace keeping missions; 7%), Desert Storm (5%), and Korea (1%), with 14% never having served in a war zone, 4% serving in multiple war zones, and 4% not responding.

### 2.2 | Measures

#### 2.2.1 | Demographic information

A brief demographic measure was utilized to collect data on veterans’ age, sex, race, marital status, and military-related factors (e.g., branch). Age, sex (coded as 1 = Male and 2 = Female), and race (coded as 1 = White and 2 = Racial/Ethnic Minority) were used as covariates in the current study.

#### 2.2.2 | Dimensional obsessive-compulsive scale (DOCS; Abramowitz et al., 2010)

The DOCS is a 20-item self-report questionnaire designed to assess the most reliably replicated dimensions of OCD. In addition to a total score, the measure yields four subscales including: contamination concerns, responsibility for harm, unacceptable thoughts, and symmetry concerns. Veterans were asked to rate each item using a 5-point Likert-type scale ranging from 0 to 4, with higher summated scores reflecting increased symptom severity in the past week. The DOCS has been found to have strong psychometric properties (Abramowitz et al., 2010). In the present study, the DOCS total score demonstrated excellent internal consistency (*α* = .95). The DOCS total score served as an independent variable in all analyses.

#### 2.2.3 | Interpersonal needs questionnaire-revised (INQ-R; Van Orden et al., 2012)

The INQ-R is a 15-item self-report questionnaire, with six items designed to assess PB and nine items designed to assess TB. Veterans read a list of questions while thinking about themselves and other people. Items were rated using a 7-point Likert-type scale ranging from 1 (*Not at all true for me*) to 7 (*Very true for me*), with higher summated scores indicating increased symptom severity. The INQ-R has been found to have strong psychometric properties (Van Orden et al., 2012). In the present study, the TB and PB scales demonstrated good to excellent internal consistency (*α* = .88 and *α* = .94, respectively). INQ-R subscales were used as the dependent variables in the current study.

#### 2.2.4 | Patient health questionnaire-9 (PHQ-9; Kroenke et al., 2001)

The PHQ-9 is a 9-item self-report questionnaire designed to assess depression diagnostic criteria and other leading major depressive symptoms. In the current study, veterans were asked to indicate how often they have been bothered by each symptom within the past 2 weeks using a 4-point Likert-type scale ranging from 0 (*Not at all*) to 3 (*Nearly every day*). Items were summed to create a total score with higher scores indicating increased symptom severity. Veterans were then dichotomized into two groups based on recommended clinical cut scores (Manea et al., 2012). Specifically, veterans scoring a 10 or above on the PHQ-9 were coded as 1 (probable depression), whereas veterans scoring a 9 or below were coded as 0 (no probable depression). The PHQ-9 has been found to have strong psychometric properties (Kroenke et al., 2001), and this method of dichotomization has been utilized in prior research (Proctor et al., 2008; Stiles-Shields et al., 2015). In the present study, the PHQ-9 demonstrated good internal consistency (*α* = .83). Probable depression was used as a covariate in the current study.

#### 2.2.5 | Trauma exposure

Veterans were verbally asked about exposure to various traumatic events (i.e., very stressful experiences involving actual or threatened death, serious injury, or sexual violence) and to identify the event that bothers them the most for the purposes of assessing posttraumatic stress disorder (PTSD) symptoms (APA, American Psychiatric Association, 2022; Frans et al., 2005). Veterans reporting exposure to an event that met Criterion A for PTSD were coded as 1, whereas those without were coded as 0. Trauma exposure was used as a covariate in all analyses.

### 2.3 | Data analytic plan

All analyses were conducted using IBM SPSS Statistics version 28. First, data screening was performed. This included calculating descriptive statistics to inspect for data entry errors, missing data, outliers, and normality. Second, descriptive statistics including means, standard deviations, and distributions (i.e., skewness and kurtosis) were examined. Third, zero-order correlations among all variables were examined. Fourth, two hierarchical regression analyses were performed to examine the association between OCD symptom severity and PB and TB (as measured by the INQ-R) after controlling for age, sex, race, trauma exposure, and probable depression (as measured by the PHQ-9). All covariates were entered into the first step of the model. The second step of the model included DOCS total scores.

## 3 | STUDY 1 RESULTS

### 3.1 | Preliminary analyses

At the item level, less than 4% of self-report data was missing. As such, missing data was handled using listwise deletion. All variables were normally distributed, with the exception of PB which was slightly skewed (i.e., statistic = 1.25, standard error = 0.24), but it was still within the normal range of −2 and 2. Given the clinical nature of the sample, no transformations were conducted on the basis of this variable. Means, standard deviations, and zero-order correlations for all variables can be found in Table 1. The mean DOCS total score was above the clinical cut-score set forth by Abramowitz et al., 2010 (i.e., >21). Further, mean levels of PB and TB were above those found in a psychiatric sample (Cero et al., 2015) as well as a sample of national guard members (Shapiro et al., 2022). A little over half of the sample (54%) reported exposure to a traumatic event consistent with Criterion A for PTSD, with 25% reporting some other stressful event or no event and 21% missing data. Finally, 60% of the sample was classified as meeting diagnostic criteria for probable depression based on PHQ-9 total scores, while 32% of the sample was classified as not meeting diagnostic criteria for probable depression (with 8% missing data).

### 3.2 | Primary analyses

The first hierarchical regression was performed to assess the relationship between OCD symptom severity and PB. Covariates were entered into the first step of the model (see Table 2), accounting for 21% of the variance in PB (*F*(5, 68) = 3.71, *p* = .005). In the second step of the model, DOCS total scores were added, accounting for an additional 6.2% of the variance (*F*_change_(1) = 5.73). Results revealed that after controlling for age (*β* = −.23, *p* = .059, *sr*^2^ = 0.04), sex (*β* = −.23, *p* = .046, *sr*^2^ = 0.04), race (*β* = −.07, *p* = .508, *sr*^2^ = 0.004), trauma exposure (*β* = .07, *p* = .562, *sr*^2^ = 0.003), and probable depression (*β* = .13, *p* = .29, *sr*^2^ = 0.01) at step 2, OCD symptom severity was significantly associated with PB (*β* = .34, *p* = .020, *sr*^2^ = 0.06).

The second hierarchical regression was performed to assess the relationship between OCD symptom severity and TB. Covariates were once again entered into the first step of the model (see Table 2), accounting for 20% of the variance in TB (*F*(5, 68) = 3.71, *p* = .010). In the second step of the model, DOCS total scores were added, accounting for an additional 7.1% of the variance (*F*_change_ (1) = 4.54) in TB. Results revealed that after controlling for age (*β* = 0.07, *p* = .569, *sr*^2^ = 0.003), sex (*β* = .04, *p* = .715, *sr*^2^ = 0.001), race (*β* = .006, *p* = .958, *sr*^2^ = 0.005), trauma exposure (*β* = .08, *p* = .522, *sr*^2^ = 0.004), and probable depression (*β* = .25, *p* = .049, *sr*^2^ = 0.05) at step 2, OCD symptom severity was significantly associated with TB (*β* = .30, *p* = 0.037, *sr*^2^ = 0.05).

## 4 | STUDY 2 METHOD

### 4.1 | Participants

Participants (*N* = 99) in the present study were recruited between 2013 and 2016, as part of a larger randomized clinical trial investigating several computerized treatments designed to target risk factors for suicide (see Allan et al., 2018; Short et al., 2019 for more information). This trial was registered at clinicaltrials.gov (#NCT01941862) before data collection. Inclusion criteria in the larger study included (1) elevated scores on one or more measures of PB (score > 9), TB (score > 21), or anxiety sensitivity cognitive concerns (score > 8; Mitchell et al., 2020; Schmidt & Joiner, 2002), (2) English speaker, and (3) being 18 years of age or older. Exclusion criteria included evidence of uncontrolled psychotic-spectrum or bipolar disorders, imminent risk of suicide necessitating hospitalization, significant medical illness, and/or participating in psychotherapy at intake. Veterans were oversampled for this study and only those identifying as a US veteran were included in the present study.

Participants ages ranged from 23 to 79 (*M* = 49.2, standard deviation [SD] = 13.01). A majority of the sample were men (*n* = 88; 88.9%) and identified as heterosexual (*n* = 97; 98%). With respect to race, 33.3% (*n* = 33) of the sample identified as White, 59.6% (*n* = 59) as Black, 1.0% (*n* = 1) as Pacific Islander, and 6.1% (*n* = 6) as another race. Five (5.1%) individuals identified as Hispanic. Additionally, 83.8% (*n* = 83) of sample met criteria for at least one psychiatric disorder. Approximately 32.3% (*n* = 32) met criteria for a primary mood disorder, 21.2% (*n* = 21) for a primary trauma-related disorder, 17.2% (*n* = 17) for a primary anxiety disorder, 7.1% (*n* = 7) for a primary substance use disorder, and 4.0% (*n* = 4) for a primary obsessive-compulsive and related disorders. Of note, an additional 5.1% (*n* = 5) of the sample met for a obsessive-compulsive and related disorder that was not primary.

### 4.2 | Procedures

All procedures in the present study were approved by the university’s institutional review board. Veterans were recruited through contacting local veteran organizations (e.g., VHA, Vet Center), flyers in the local community, newspaper advertisements, website listings, and community mail-outs to local medical and mental health providers. Interested participants were contacted via phone to complete a brief phone interview. If participants met initial criteria, they were invited to an in-person screening appointment. Suicide risk was assessed at all timepoints via interview and self-report measures. Actions were taken to provide the appropriate care based on designated risk (e.g., suicide hotline information, creating a safety plan, means restriction; Chu et al., 2015). At the initial screening appointment, participants completed a diagnostic interview (i.e., *Structured Clinical Interview for DSM-5, Research Version*) administered by trained doctoral students and self-report measures. Participants meeting inclusion criteria were randomized to one of four treatment conditions (see Intervention Conditions section) using a random number generator (for more information and consort diagram see Allan et al., 2018, 2020). During the intervention phase, participants received their assigned treatment at a rate of one session (approximately 60 min) per week for 3 weeks. During each session, participants completed their assigned intervention followed by self-report measures. Participants were then scheduled for their next assessment to occur approximately 1-month after treatment. Of note, the larger study collected additional data at 3, 6, and 12 months after treatment (Allan et al., 2018; Short et al., 2019).

### 4.3 | Intervention conditions

Since the effect of treatment is not the primary aim of this present investigation, a brief description of conditions can be found below (for more detailed information on treatment conditions see Allan et al., 2018; Short et al., 2019). Individuals in the larger study were randomly assigned to either an anxiety intervention condition, mood intervention condition, a combined anxiety and mood condition, or a repeated contact control. The anxiety intervention condition included a Cognitive Anxiety Sensitivity Treatment (CAST; Schmidt et al., 2014) and an anxiety focused cognitive bias modification program (CBM-I; Capron & Schmidt, 2016). CAST is a computerized intervention which provides educational and behavioral techniques that are commonly used in anxiety treatments. CBM-I is a computerized treatment where individuals are presented with a word for 1 s (e.g., “excited”) followed by a sentence (e.g., “You notice your heart is beating faster”) and asked to judge if the word and sentence were related with half of the trials having a combination that could be interpreted as benign and the other half as anxious. The mood condition was consistent with the anxiety condition where participants completed a top-down psychoeducational portion as well as a bottom-up CBM-I portion. The combined condition consisted of both the anxiety and mood condition. Finally, the repeated contact control condition consisted of participants being assigned a clinician on the research staff that would call them once a week for a brief suicide risk check-in.

### 4.4 | Measures

#### 4.4.1 | Demographic information

All demographic information was collected through self-report. Information on age, sex, sexual orientation, and race were all collected and assessed similar to Study 1.

#### 4.4.2 | Dimensional obsessive-compulsive scale (DOCS; Abramowitz et al., 2010)

See Study 1 for a full description of the DOCS. In the present study, the DOCS total score demonstrated excellent internal consistency (*α* = .97).

#### 4.4.3 | Interpersonal needs questionnaire-revised (INQ-R; Van Orden et al., 2012)

See Study 1 for a full description of the INQ-R. In the present study, the TB and PB scales demonstrated excellent internal consistency (*α* = .91 and *α* = .94, respectively).

#### 4.4.4 | Structured clinical interview for DSM-5, research version (SCID-5-RV; First et al., 2015)

The SCID-5, a well validated semi-structured interview, was used to assess the presence of major DSM-5 diagnoses including major depressive disorder, and the presence of a Criterion A trauma for the purposes of assessing posttraumatic stress disorder. All interviews were conducted by highly trained doctoral students and reviewed with a licensed clinical psychologist during a weekly supervision meeting.

#### 4.4.5 | Depressive symptom index—suicidality subscale (DSI-SS; Joiner et al., 2002)

The DSI-SS is a 4-item self-report measure of suicidal ideation severity (i.e., ideation, suicidal plans, control of suicidal thoughts, and suicidal impulses) over the past 2 weeks. Participants were asked to rate each item on 4-point Likert-type scale ranging from 0 (*None*) to 3 (*Always*). A total score is calculated by adding each item with higher scores being reflective of more severe suicidal ideation. The DSI-SS has demonstrated strong psychometric properties in both research and clinical settings (Joiner et al., 2002; Stanley et al., 2021). The internal consistency of the DSI-SS in the present study was excellent (α= 0.92).

### 4.5 | Data analytic plan

All analyses in the present study were conducted using *R* version 4.2.2 (R Core Team, 2020). Before main analyses, data were screened for missingness, normality, and multicollinearity. After preliminary analyses and data screening, the direct effects of OCD symptoms at baseline on suicidal ideation severity at 1-month follow-up covarying for treatment condition was conducted using robust maximum likelihood (i.e., full information maximum likelihood with the Yuan-Bentler scaled chi-square index [Y-B χ^2^]) was used to adjust standard errors for data nonnormality and address missing data. Mediation analyses were then conducted to determine the indirect effect of OCD symptoms on suicidal ideation severity via PB and TB. Structural equation modeling through the lavaan package (Rosseel, 2012) was used to test this effect using MacKinnon’s approach to mediation (MacKinnon et al., 2002). We constructed a path model examining the effect of baseline OCD symptoms on the mediator at post-treatment (Path *a*) and the effect of the mediator on suicidal ideation severity at 1-month follow-up (Path *b*) independent of the effect of OCD symptoms (Path *c*’). The indirect effect of OCD symptoms on suicidal ideation severity was calculated as the product of paths *a* and *b*. To test the statistical significance of the mediation, bootstrapped confidence intervals around the indirect effect were calculated using bootstrapped resampling (Preacher & Hayes, 2008) where an effect is significant when the confidence interval does not include zero. In the present analyses, 5000 resamples were used to generate the 95% confidence intervals.

## 5 | STUDY 2 RESULTS

### 5.1 | Preliminary analyses

Before any analyses, data were screened for missingness, normality, and multicollinearity. Missing data patterns were analyzed, and the data were determined to be missing at random. Full information maximum likelihood estimation was used to account for missing data. The mean OCD symptom severity score was 22.85 (SD = 17.41) at baseline. Participants endorsed above clinical threshold scores on PB (*M* = 13.28, SD = 10.56) and TB (*M* = 35.10, SD = 14.38) at post-treatment. The mean suicidal ideation severity score was 0.67 (SD = 1.62). A total of 14 (14.1%) veterans met current criteria for major depressive disorder, and 74 (47.7%) veterans reported experiencing a Criterion A trauma. Most of data were found to not violate assumptions of normality (i.e., skewness and kurtosis) and multicollinearity, but the suicidal ideation severity score was slightly skewed and leptokurtic. Thus, all analyses with suicidal ideation used robust maximum likelihood to account for nonnormality.

### 5.2 | Direct effects

Before examining the indirect effects of OCD symptom severity at baseline on 1-month follow-up suicidal ideation via PB and TB, the direct effect of OCD symptom severity on suicidal ideation at follow-up were examined. OCD symptom severity was found to significantly predict suicidal ideation at follow-up (*β* = .24, *p* = .023). Further, this relationship remained (*β* = .23, *p* = .028) with the inclusion of treatment condition (*β* = −.02, *p* = .88) as a covariate. Similarly, OCD symptom severity was found to significantly predict post-treatment PB (*β* = .33, *p* < .001) and TB (*β* = .26, *p* = .010) after covarying for treatment condition (PB model: *β* = .17, *p* = .087; TB model: *β* = .01, *p* = .943) in both models respectively.

### 5.3 | Mediation models

**FIGURE 1:**
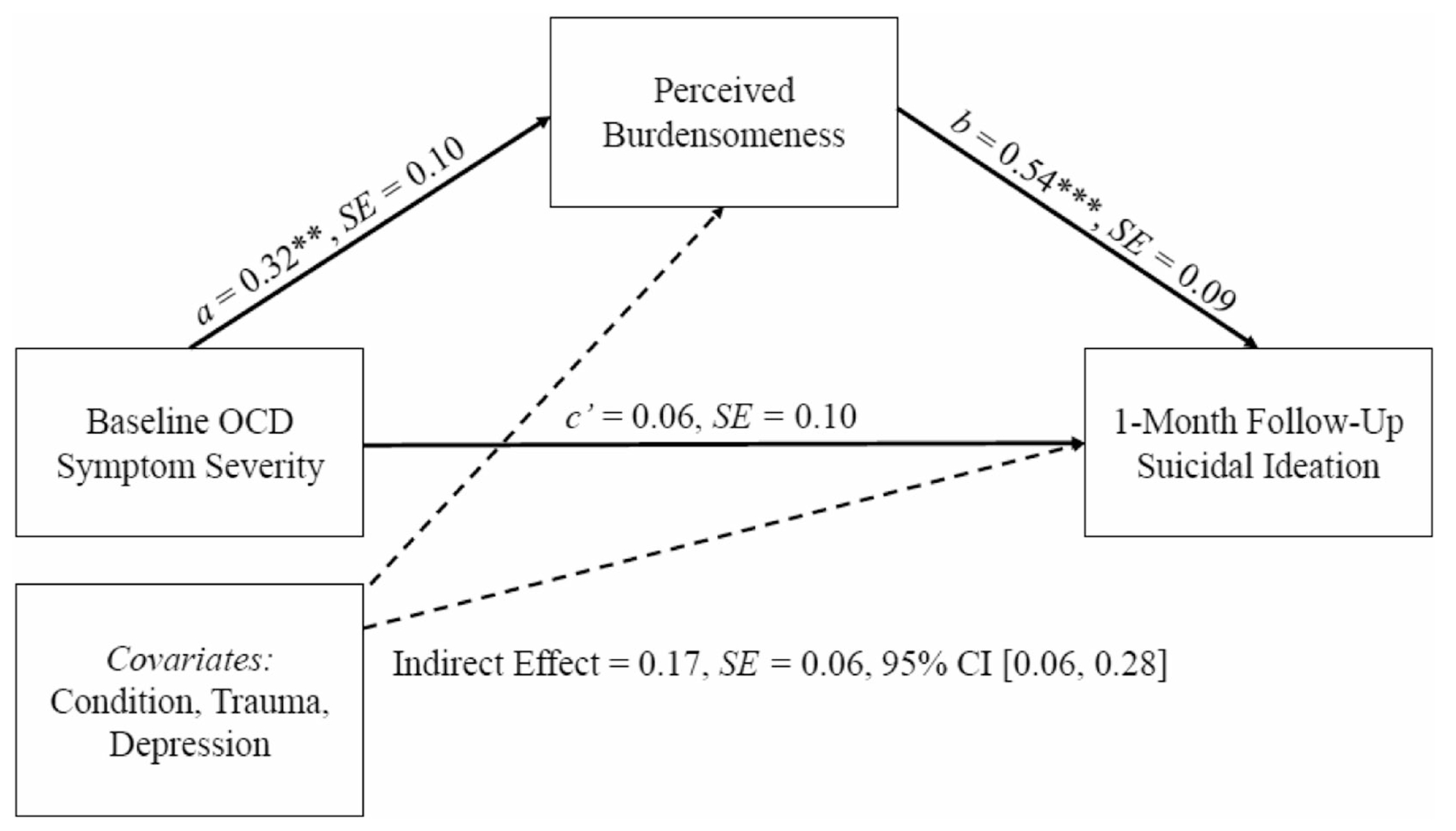
Standardized path coefficients for indirect effect of baseline OCD symptom severity on 1-month follow-up suicidal ideation via perceived burdensomeness at post-treatment. *p < .05, **p < .01, ***p < .001. OCD, obsessive-compulsive disorder.

**FIGURE 2:**
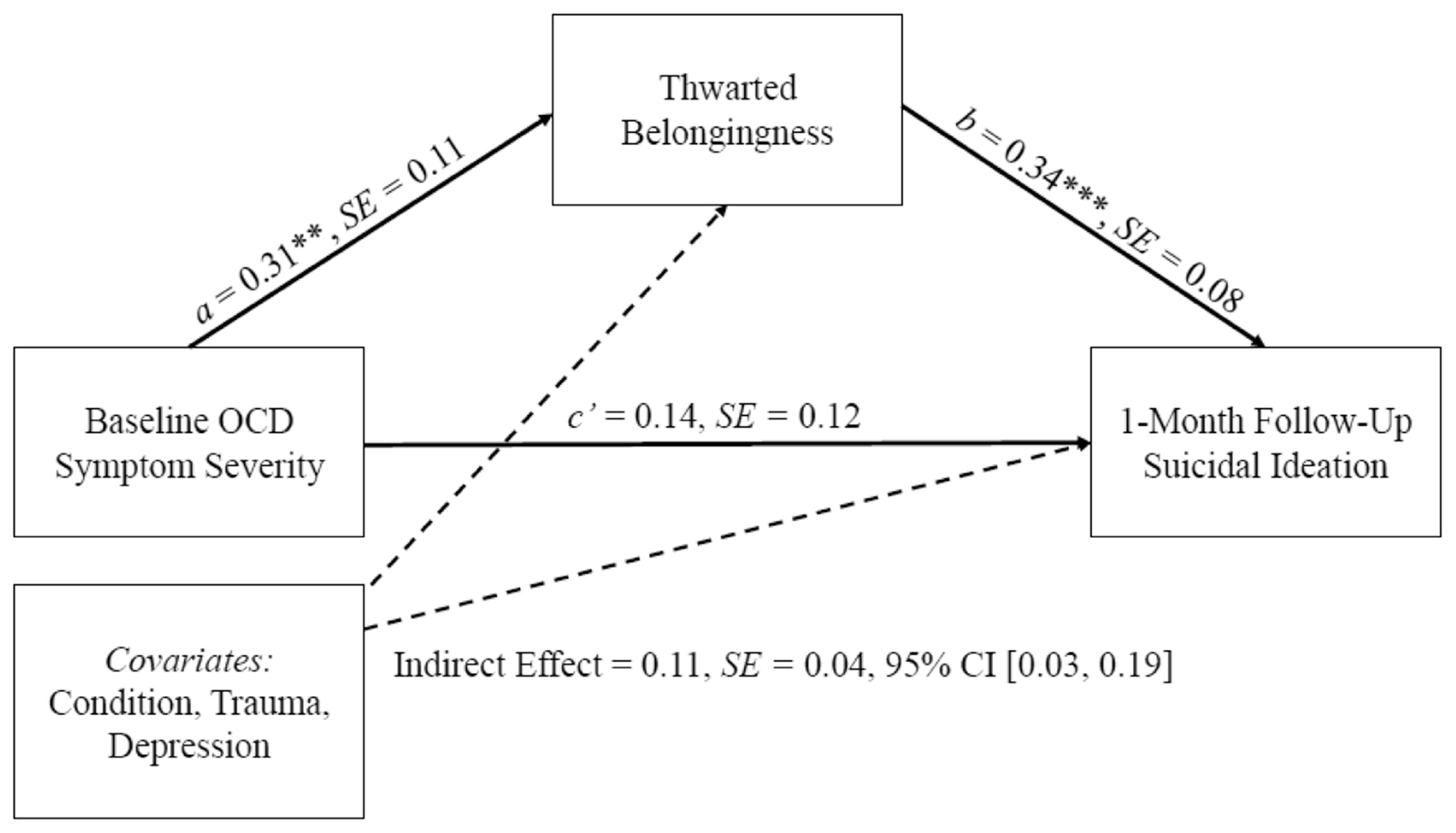
Standardized path coefficients for indirect effect of baseline OCD symptom severity on 1-month follow-up suicidal ideation via thwarted belongingness at post-treatment. *p < .05, **p < .01, ***p < .001. OCD, obsessive-compulsive disorder.

The standardized path estimates for both mediation models can be found in Figures 1 and 2. There was a significant indirect effect of OCD symptom severity on suicidal ideation via post-treatment PB (*b* = 0.02, SE = 0.01, *β* = .25, 95% confidence interval [CI] [0.06–0.31], *p* = .003). With the inclusion of the indirect path, the direct effect (*c*’ path) of OCD symptom severity on suicidal ideation was no longer significant (*b* = 0.006, SE = 0.09, *β* = .07, *p* = .495). Further, there was also a significant indirect effect of OCD symptom severity on suicidal ideation via post-treatment TB (*b* = 0.02, SE = 0.01, *β* = .11, 95% CI [0.03–0.19], *p* = .010). Similarly, with the inclusion of this mediating path, the direct effect (*c*’ path) of OCD symptom severity on suicidal ideation was no longer significant (*b* = 0.01, SE = 0.01, *β* =.14, *p* =.220).

## 6 | DISCUSSION

The goal of the present study was to explore both the cross-sectional and longitudinal relationships between OCD symptoms, PB, TB, and suicidality among US veterans. Consistent with our hypotheses, we found that that OCD symptoms were significantly associated with PB and TB at the cross-sectional level in a sample of treatment-seeking veterans. Further, we found this relationship remained significant while adjusting for demographic factors, trauma exposure, and probable depression. These findings suggest that symptoms of OCD may play a unique role in both PB and TB in veterans. With both PB and TB being identified as key targetable risk factors of suicide (Chu et al., 2017), these findings provide rationale that OCD symptoms may contribute to both factors above and beyond other key factors (e.g., trauma, depression) that are regularly screened for in treatment settings for veterans.

Further consistent with our hypotheses, we found that OCD symptoms were significantly related to both PB and TB as well as suicidal ideation longitudinally. Given that PB and TB have been found to be risk factors for suicide, we also sought to determine if the relationship between OCD and suicidal ideation were mediated by PB and TB. Using three timepoints, we found that OCD is indirectly related to suicidal ideation via PB and TB while covarying for trauma exposure and depression diagnosis. Notably, the direct effect of OCD on suicidal outcomes was no longer significant with the inclusion of the indirect effect of PB and TB. These findings suggest that veterans with greater OCD symptoms subsequently experience greater levels of PB and TB which in turn leads to increased severity in suicidal ideation. These findings are consistent with the interpersonal theory of suicide and previous studies that have demonstrated PB and TB mediating the effect between factors such as anxiety, depression, and posttraumatic stress disorder with suicide in civilian populations (Hill et al., 2018; Jahn et al., 2011; Poindexter et al., 2015). Future studies should seek to explore why OCD symptoms lead to greater PB and TB to further bolster efforts to treat these phenomena in veterans. Factors such as interpersonal skills deficits (Jansen et al., 2020) and accommodation of others due to OCD (Lebowitz et al., 2012) could explain the particular relationships with PB and TB that may warrant further study. Further, future research should explore whether these relationships look different in the general civilian population. Given the unique factors associated with being a veteran (e.g., combat exposure, deployment to war zones, and rank) it is possible that these relationships may present differently in the general population (Phillips et al., 2010; Xue et al., 2015).

Taken together, the present investigation provides evidence that among veterans OCD symptoms should be of greater concern for treatment providers. While depression and posttraumatic stress disorder remain essential treatment targets in the VA, the present findings highlight the importance of assessing for and treating OCD within this population. Left alone, OCD may increase risk for suicide within veterans. It is vital that symptoms of OCD are not only better understood in veterans but also addressed because they are related to suicidal ideation. The present study also highlights the need for treatments that can directly target PB and TB within veterans (Short et al., 2020). Future treatments could focus on targeting feelings of loneliness due to the conceptual overlap with TB (Cacioppo et al., 2015). Similarly, treatments could focus on targeting social support to combat effects of PB (Hogan et al., 2002; Lakey, 2010). Treatments that target these mechanisms would provide a parsimonious solution to address suicide risk within the veteran population regardless of diagnosis of OCD, depression, or posttraumatic stress disorder. At the same time, veteran treatment settings such as the VHA should increase efforts to screen for OCD to ensure proper care is provided (Ennis et al., 2022).

The findings of this investigation should be considered in the context of limitations. First, while mean OCD symptom severity scores across both studies were above clinically meaningful thresholds, diagnostic data was not available for Study 1 and only nine veterans in Study 2 were diagnosed with OCD. Future studies should examine these relationships within a sample of veterans with OCD to further parse these relationships. However, the present study provides evidence that symptoms of OCD may still be vital to assess and treat in veterans despite the lack of diagnosis. Second, the sample size of both studies limited the ability to vary for additional variables that may better contextualize these relationships. Third, while both studies were able to covary for trauma exposure and depression, more dimensional measures of PTSD and depression may reveal more nuanced relationships between these variables. Future studies should seek to replicate these findings in larger veteran samples using symptom severity measures to further parse these relationships. While it was not the aim of the study, we also were not able to assess suicidal ideation in the first sample allowing for replication of certain relationships. Finally, the endorsement of suicidal ideation was low in Study 2, and these findings should be explored in a higher risk sample.

In the present investigation, we examined secondary data from two separate veteran samples to identify the relationships between OCD, PB, TB, and suicidal ideation. We found that OCD was uniquely related to both PB and TB, and that the association between OCD and suicidal ideation was indirectly explained by both PB and TB. Altogether, the present study adds to vital research on veteran suicide demonstrating the importance of assessing for and addressing OCD and its symptoms.

## Supplementary Material

Supplemental Material

Additional supporting information can be found online in the Supporting Information section at the end of this article.

## Figures and Tables

**TABLE 1:** Study 1—Means, standard deviations, and zero order correlations for all variables.

	Variables	1	2	3	4	5	6	7	8
1.	Age	–							
2.	Sex	−0.38**	–						
3.	Race	−0.14	0.19	–					
4.	Trauma Exposure	0.07	−0.02	0.04	–				
5.	PHQ-9 Depression	−0.11	−0.02	0.15	0.12	–			
6.	DOCS - Total Score	−0.30**	0.16	0.28**	0.38**	0.49**	–		
7.	INQ-R - PB Subscale	−0.24*	−0.11	0.03	0.19	0.31**	0.43**	–	
8.	INQ-R - TB Subscale	−0.06	0.06	0.13	0.23	0.40**	0.44*	0.39**	–
	*M*	54.29	–	–	–	–	31.71	13.49	34.99
	SD	14.95	–	–	–	–	15.99	9.24	12.04

*Note*: Sex coded as 1 = Male and 2 = Female, Race coded as 1 = White and 2 = Racial/Ethnic Minority, Trauma Exposure coded as 1 = Yes and 0 = No.

Abbreviations: DOCS-Total Score, Dimensional Obsessive-Compulsive Scale Total Score; INQ-R-PB Subscale, Interpersonal Needs Questionnaire-Revised Perceived Burdensomeness Subscale; INQ-R-TB Subscale, Interpersonal Needs Questionnaire-Revised Thwarted Belongingness Subscale; PHQ-9 Depression, Patient Health Questionnaire-9 probable Depression coded as 1 = Yes and 0 = No.

** *p* <.01;

* *p* < .05.

**TABLE 2:** Summary of Hierarchical Linear Regression Analyses Predicting PB and TB (*N* = 100).

Variable	Model 1—perceived burdensomeness					
	*b*	SE	*β*	*t*	*p* Value	*sr* ^2^
Step 1 (*R*^2^ = 0.21)					.005	
Age	−0.19	0.07	−0.31	−2.64	.010	0.081
Sex	−5.17	2.81	−0.22	−1.84	.070	0.039
Race	−0.37	2.07	−0.02	−0.18	.857	0.001
Trauma Exposure	3.61	2.15	0.18	1.68	.097	0.033
PHQ-9 Depression	4.99	2.13	0.26	2.34	.022	0.064

Step 2 (ΔR^2^ = 0.06)					.020	
DOCS	0.19	0.08	0.34	2.39	.020	0.06
	Model 2 —Thwarted Belongingness					
Variable	*b*	SE	*β*	*t*	*p* Value	*sr* ^2^

Step 1 (*R*^2^ = 0.20)					.010	
Age	−0.01	0.10	−0.01	−0.05	.960	0.000
Sex	1.73	3.70	0.06	0.47	.642	0.002
Race	1.31	2.73	0.05	0.48	.633	0.002
Trauma Exposure	4.65	2.83	0.18	1.64	.105	0.032
PHQ-9 Depression	9.21	2.81	0.37	3.28	.002	0.127

Step 2 (Δ*R*^2^ = 0.07)					.037	
DOCS	0.23	0.11	0.30	2.13	.037	0.071

Abbreviations: *b*, Unstandardized regression coefficient; DOCS, Dimensional Obsessive-Compulsive Scale Total Score; INQ-R-PB Subscale, Interpersonal Needs Questionnaire-Revised Perceived Burdensomeness Subscale; INQ-R-TB Subscale, Interpersonal Needs Questionnaire-Revised Thwarted Belongingness Subscale; SE, Standard Error; *sr*^2^, semi-partial *r* squared; *β*, Standardized regression coefficient; Δ*R*^2^, change in *R*^2^.

## Data Availability

The data that support the findings of this study are available on request from the corresponding author. The data are not publicly available due to privacy or ethical restrictions.

## Footnotes

1 CBOCs were established by Congress in the 1990s to improve access to care for veterans living in rural and underserved areas. Currently, there are over 800 clinics throughout the United States and its territories.
