# Supplementary material for: Obsessive-compulsive symptoms, perceived burdensomeness, and thwarted belongingness: Associations and implications among US veterans
Source: J Clin Psychol. Author manuscript; Available in PMC 2026 Jun 2. (PMC13229094; doi:10.1002/jclp.23609)
Supplement: Supplemental Material [file NIHMS2174602-supplement-Supplemental_Material.docx]

**Supplemental Table 1**

*Study 2—Means, standard deviations, and zero order correlations for all variables*

|  | Variables | 1 | 2 | 3 | 4 | 5 | 6 | 7 | 8 | 9 | 10 | 11 | 12 |
| --- | --- | --- | --- | --- | --- | --- | --- | --- | --- | --- | --- | --- | --- |
|  | Baseline |  |  |  |  |  |  |  |  |  |  |  |  |
| 1. | DOCS - Total Score | ­­–– |  |  |  |  |  |  |  |  |  |  |  |
| 2. | INQ-R - PB Subscale | .48*** | –– |  |  |  |  |  |  |  |  |  |  |
| 3. | INQ-R - TB Subscale | .32** | .63*** | –– |  |  |  |  |  |  |  |  |  |
| 4. | DSI-SS | .29** | .48*** | .40*** | –– |  |  |  |  |  |  |  |  |
|  | Posttreatment |  |  |  |  |  |  |  |  |  |  |  |  |
| 5. | DOCS - Total Score | .72*** | .40*** | .28** | .22* | –– |  |  |  |  |  |  |  |
| 6. | INQ-R - PB Subscale | .34** | .49*** | .32** | .44*** | .53*** | –– |  |  |  |  |  |  |
| 7. | INQ-R - TB Subscale | .26* | .46*** | .62*** | .46*** | .37*** | .57*** | –– |  |  |  |  |  |
| 8. | DSI-SS | .25* | .29** | .21* | .64*** | .28** | .51*** | .40*** | –– |  |  |  |  |
|  | 1-Month Follow-up |  |  |  |  |  |  |  |  |  |  |  |  |
| 9. | DOCS - Total Score | .72*** | .42*** | .30** | .40*** | .78*** | .49*** | .36*** | .30** | –– |  |  |  |
| 10. | INQ-R - PB Subscale | .35** | .58*** | .40*** | .52*** | .52*** | .86*** | .56*** | .40*** | .54*** | –– |  |  |
| 11. | INQ-R - TB Subscale | .31** | .47*** | .63*** | .37*** | .28** | .36*** | .74*** | .16 | .39*** | .49** | –– |  |
| 12. | DSI-SS | .23* | .39*** | .31** | .77*** | .20 | .55*** | .37*** | .77*** | .36*** | .60** | .31** | –– |
|  | *M* | 22.85 | 15.67 | 37.24 | 1.00 | 18.05 | 13.28 | 35.1 | 0.59 | 16.61 | 12.50 | 35.6 | 0.67 |
|  | *SD* | 17.41 | 10.56 | 14.02 | 1.84 | 14.92 | 8.91 | 14.38 | 1.40 | 15.52 | 8.75 | 14.38 | 1.62 |
| *Note.* DOCS - Total Score = Dimensional Obsessive-Compulsive Scale Total Score, INQ-R - PB Subscale = Interpersonal Needs Questionnaire-Revised Perceived Burdensomeness Subscale, INQ-R - TB Subscale = Interpersonal Needs Questionnaire-Revised Thwarted Belongingness Subscale.  *** *p* <.001, ***p* < .01, * *p* < .05. | | | | | | | | | | | | | |
